# Fatty Acid Trafficking Between Lipid Droplets and Mitochondria: An Emerging Perspective

**DOI:** 10.7150/ijbs.105361

**Published:** 2025-02-10

**Authors:** Katarína Smolková, Klára Gotvaldová

**Affiliations:** Laboratory of Mitochondrial Physiology, Institute of Physiology of the Czech Academy of Sciences.

**Keywords:** lipid droplets, mitochondria, CPT1, triglycerides, lipotoxicity, ferroptosis

## Abstract

The current understanding of lipid droplets (LDs) in cell biology has evolved from being viewed merely as storage compartments. LDs are now recognized as metabolic hubs that act as cytosolic buffers against the detrimental effects of free fatty acids (FAs). Upon activation, FAs traverse various cellular pathways, including oxidation in mitochondria, integration into complex lipids, or storage in triacylglycerols (TGs). Maintaining a balance among these processes is crucial in cellular FA trafficking, and under metabolically challenging circumstances the routes of FA metabolism adapt to meet the current cellular needs. This typically involves an increased demand for anabolic intermediates or energy and the prevention of redox stress. Surprisingly, LDs accumulate under certain conditions such as amino acid starvation. This review explores the biochemical aspects of FA utilization in both physiological contexts and within cancer cells, focusing on the metabolism of TGs, cholesteryl esters (CEs), and mitochondrial FA oxidation. Emphasis is placed on the potential toxicity associated with non-esterified FAs in cytosolic and mitochondrial compartments. Additionally, we discuss mechanisms that lead to increased LD biogenesis due to an inhibited mitochondrial import of FAs.

## 1. Introduction

Lipid droplets (LDs) are organelles specialized in storing and releasing FAs. The main lipid species in LDs are triglycerides (TGs) and cholesteryl-esters (CEs). LDs are formed in response to an elevated uptake of substrates, namely free fatty acids (FAs) and low-density lipoproteins (LDLs), or the induction of TG or CE synthesis. LDs, once considered inert repositories of lipids and energy, have emerged as dynamic organelles with multifaceted roles in lipid homeostasis and signaling. Via the synthesis of TGs and CEs and their storage in an inert compartment, LDs buffer free FAs to prevent lipotoxicity, which typically refers to the harmful effects of excessive levels of certain FAs in the cell. The lipotoxic effects of both saturated and unsaturated FAs confer mitochondrial dysfunction, endoplasmic reticulum (ER) stress, oxidative stress, and possibly also impairment of membrane integrity resulting in ferroptosis. We aim to summarize the nuanced relationship between LDs and lipotoxicity, focusing specifically on mitochondrial and cytosolic milieus.

Cancer cells and tissues are valuable models for studying cellular metabolic processes. Given the roles of TGs and CEs in LD biogenesis, we ask how the principles of LD biology translate into cancer metabolism. An increased accumulation of LDs has been detected in human cancer tissues in colorectal, renal, prostate cancers, or breast carcinoma resistant to tamoxifen 1-4. LD accumulation has been traditionally recognized and studied in clear cell renal carcinoma and prostate cancer, and several enzymes responsible for TG or CE synthesis in cancer tissues have been associated with a progressive cancer phenotype. Therefore, if the LD-forming apparatus is systematically upregulated in some cancer indications, we ask what the consequences for cancer growth are, and which mechanisms related to LD biogenesis or lipotoxicity are targetable for cancer growth.

LDs are no longer only studied in adipose tissues, and in non-adipose tissues there are recognized circumstances that contribute to LD biogenesis, such as the onset of autophagy or mitochondrial defects associated with an inhibited mitochondrial oxidation of FAs. There are some critical gaps in our understanding of FA trafficking that could shed light on the pathophysiological mechanisms underlying various metabolic disorders that are associated with LD build-up. The exploration of LD biology in the context of inter-organelle crosstalk promises insights that extend beyond metabolic processes and provides novel perspectives for addressing a wide range of pathophysiological conditions, such as type 2 diabetes, cancer and associated complications, such as cancer cachexia 5.

## 2. The role of DGAT1/2 in TG handling and LD formation

**Figure 1:**
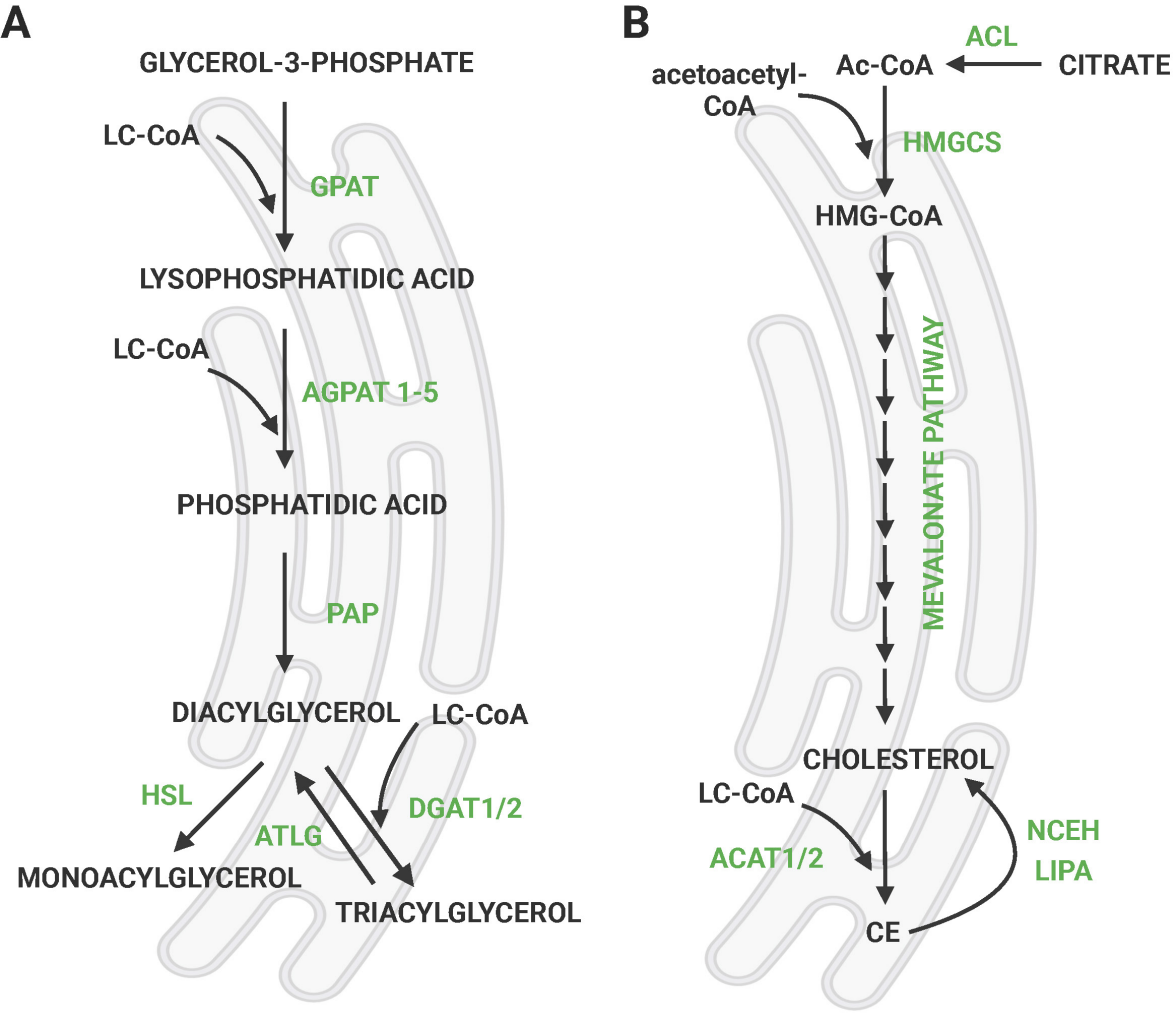
Synthesis of triglyceride and cholesteryl esters. **a**) Scheme of *de novo* synthesis of TGs via glycerol phosphate pathway. G3P is imported into the ER, and the remaining steps take place there. LDs are formed from the ER membrane via a budding mechanism. **b**) Mevalonate (isoprenoid) pathway of cholesterol synthesis followed by esterification with FAs. Citrate exported from mitochondria is converted into acetyl-coA, which is a precursor of cholesterol. The pathway takes place in the ER. Created with Biorender.com. *Abbreviations*: **ACAT:** acetyl-CoA acetyltransferase; **ACL:** ATP-citrate lyase; **Ac-CoA:** acetyl-CoA; **AGPAT:** 1-acylglycerol-3-phosphate O-Acyltransferase; **ATGL:** adipose triglyceride lipase; **CE:** cholesteryl ester;** DGAT:** acyl-CoA:diacylglycerol acyltransferase; **GPAT:** glycerol-3-Phosphate acyltransferase; **HMG-CoA:** hydroxymethylglutaryl-CoA; **HMGCS:** hydroxymethylglutaryl-CoA synthase; **HSL:** hormone-sensitive lipase; **LC-CoA:** long-chain CoA; **LIPA:** lysosomal acid lipase/cholesteryl ester hydrolase; **NCEH:** neutral cholesterol ester hydrolase 1; **PAP:** phosphatidic acid phosphatase.

*De novo* LD formation involves the synthesis of TGs occurring between two leaflets of the ER membrane 6 by the so-called budding mechanism 7. This process results in the accumulation and separation of discrete LDs in the cytosol 8, which are further enlarged 9. TGs are synthesized through the glycerol phosphate pathway (Fig. 1a), which involves several sequential enzymatic reactions starting from glycerol-3-phosphate (G3P) into diacylglycerol (DG) 10. The final, rate-limiting step in TG synthesis is the esterification of DGs, catalyzed by two enzymes; acyl CoA:diacylglycerol acyltransferase (DGAT) 1 or DGAT2 11. DGAT1 and DGAT2 share no sequence similarity and localize to different cellular compartments; DGAT1 is an exclusive ER enzyme, whereas DGAT2 also localizes to LDs 9, 12. The adipose tissue-specific double knockout (KO) model suggested that there is an alternative pathway of TG synthesis apart from DGAT1 and DGAT2 13. The acyltransferase transmembrane protein 68/DIESL was recently identified, which catalyzes DG esterification from acyl donors other than activated FAs, such as phospholipids (PLs) 14. However, the synthesis of TGs via DGAT1/2 remains the major contributor to TG synthesis in the LD pool.

Extensive studies of DGAT1 and DGAT2 in several models of total KO, conditional KO, or mouse embryonal fibroblasts (MEFs) have contributed to our understanding of their functional role in physiology and during metabolic dysbalance. TG levels were severely affected in DGAT1 KO in adipose and non-adipose tissues, essentially when mice were challenged with a high-fat diet (HFD) 15. The evidence suggests that DGAT variants cannot fully compensate for their function in every physiological aspect. Early reports demonstrated that DGAT1 is not essential for survival, while DGAT2 deletion is lethal postnatally due to the impaired permeability barrier function in the skin and serious defects in energy metabolism 16, 17. Either DGAT1 KO or DGAT2 KO-derived MEFs were still able to form LDs, however, only the genetic deletion of both enzymes simultaneously results in minimizing TG synthesis; instead, exogenous FAs were incorporated into PLs (such as phosphatidylcholine, PC, or phosphatidylserine, PS) 11.

Another fact demonstrated collectively is that DGAT1 esterifies DGs with FAs coming from dietary lipids, while DGAT2 works alongside *de novo* lipogenesis, which is the most obvious in tissues specialized for the synthesis of TG, i.e. in adipose tissue and in the liver. Using adipose tissue-specific KO animals 17, it was shown that DGAT1 and DGAT2 can substitute for each other's functions when fed with standard chow, but an HFD induced marked metabolic changes in adipose tissue-specific DGAT1 KO, such as a reduction in fat mass compared to adipose-specific DGAT2 KO. This was interpreted as the preferred channeling of dietary lipids into the DGAT1 reaction. Moreover, DGAT2 could not compensate for the loss of DGAT1 in adipose tissue, demonstrated by the fact that DGAT1, but not DGAT2 deletion in combination with a HFD leads to ER stress, lipotoxicity, and systemic physiological changes such as insulin resistance 17. In the liver, likewise, DGAT1 preferentially utilizes exogenous FAs for TG synthesis, since the deletion of hepatic DGAT1 protected against liver steatosis from an HFD, but did not protect against steatosis induced by *de novo* lipogenic substrates 18. Likewise, DGAT2 deletion in the liver led to a massive decrease in TG and DG levels when fed by an FPC diet (fructose, palmitate, cholesterol, and trans fat) containing *de novo* lipogenic substrates (i.e. G3P) 19. On the other hand, in enterocytes, cell types critical for dietary fat absorption and handling, DGAT1 and DGAT2 also fulfill different functions. While the overexpression of any DGAT enzyme upregulated TGs in the ER, only DGAT2 overexpression contributed to a higher release of TG and chylomicrons into the bloodstream 20. In summary, the role of DGAT1 and DGAT2 differ, especially under pathophysiological conditions. Substrate specificity and time resolution studies are needed to explain the key biochemical differences associated with TG synthesis in distinct tissues.

**Figure 2:**
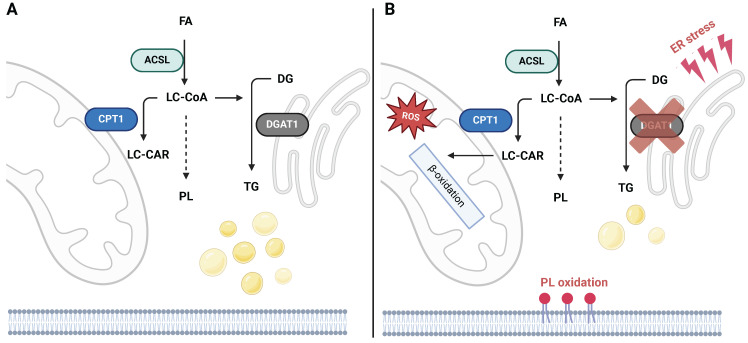
Mechanism of cellular toxicity induced by DGAT1 inhibition. **a**) LC-CoAs are distributed between mitochondrial oxidation, PL synthesis, and TG synthesis. **b**) Several studies indicate that the inhibition of DGAT1 induces severe toxicities related to oxidative stress, which stem from the redirection of activated FAs into other metabolic routes. These include augmented mitochondrial FA import and related ROS production, synthesis of PL species sensitive to oxidation and ferroptosis initiation, and ER stress related to the imbalance of biosynthetic processes in the ER. Created with BioRender.com. *Abreviations*: **ACSL:** long-chain-fatty-acid-CoA ligase; **CPT1:** carnitine palmitoyltransferase 1; **DG:** diacylglycerol; **DGAT:** acyl-CoA:diacylglycerol acyltransferase; **FA:** fatty acid; **LC-CAR:** long-chain carnitine; **LC-CoA:** long-chain CoA; **PL:** phospholipid; **TG:** triacylglycerol.

The principles of TG synthesis regulation also translate into cancer biology in several unrelated malignancies. An analysis of public genomic datasets revealed that high DGAT1 levels in melanoma patients were associated with worse progression-free survival 21, as was also shown using the glioblastoma multiforme (GBM) dataset 22. Another study reported a positive correlation between DGAT1 and the LD pool in prostate cancer *in vitro*, and that DGAT1 knockdown suppresses colony formation 23. A positive association of DGAT1 and cancer growth was confirmed *in vivo* in zebrafish melanocytes 21, in which DGAT1 overexpression induced melanoma growth, and also in a xenograft model of GBM in mice, in which both genetic and pharmacological suppression of DGAT1 inhibited GBM growth 22. Both studies also point to mitochondria-related toxicity as a consequence of DGAT1 inhibition (DGATi) and the mechanism of DGATi-related tumor suppression. In principle, DGAT1-induced LD formation prevents FA accumulation in mitochondria or in PLs sensitive to oxidation (PE, and PC containing polyunsaturated fatty acids, PUFAs) 21. DGAT1i induced FA oxidation, leading to excessive reactive oxygen species (ROS) production, mitochondrial damage, and 4-hydroxynonenal accumulation, which could all be prevented with N-acetyl-cysteine treatment. Aside from mitochondrial damage, a redistribution of PUFA-containing PLs, ER stress markers, and ceramide accumulation was detected after the inhibition of LD biogenesis 21, 22. These observations suggest that LDs in cancer tissues could participate in preventing lipotoxicity; DGAT1 reaction and LD biogenesis can be perceived as a sink for non-esterified FAs, which in turn protects from the lipotoxic effect of FA oxidation, or its incorporation into oxidation-sensitive PLs (Fig. 2).

## 3. CE synthesis and ACAT biology

**Figure 3:**
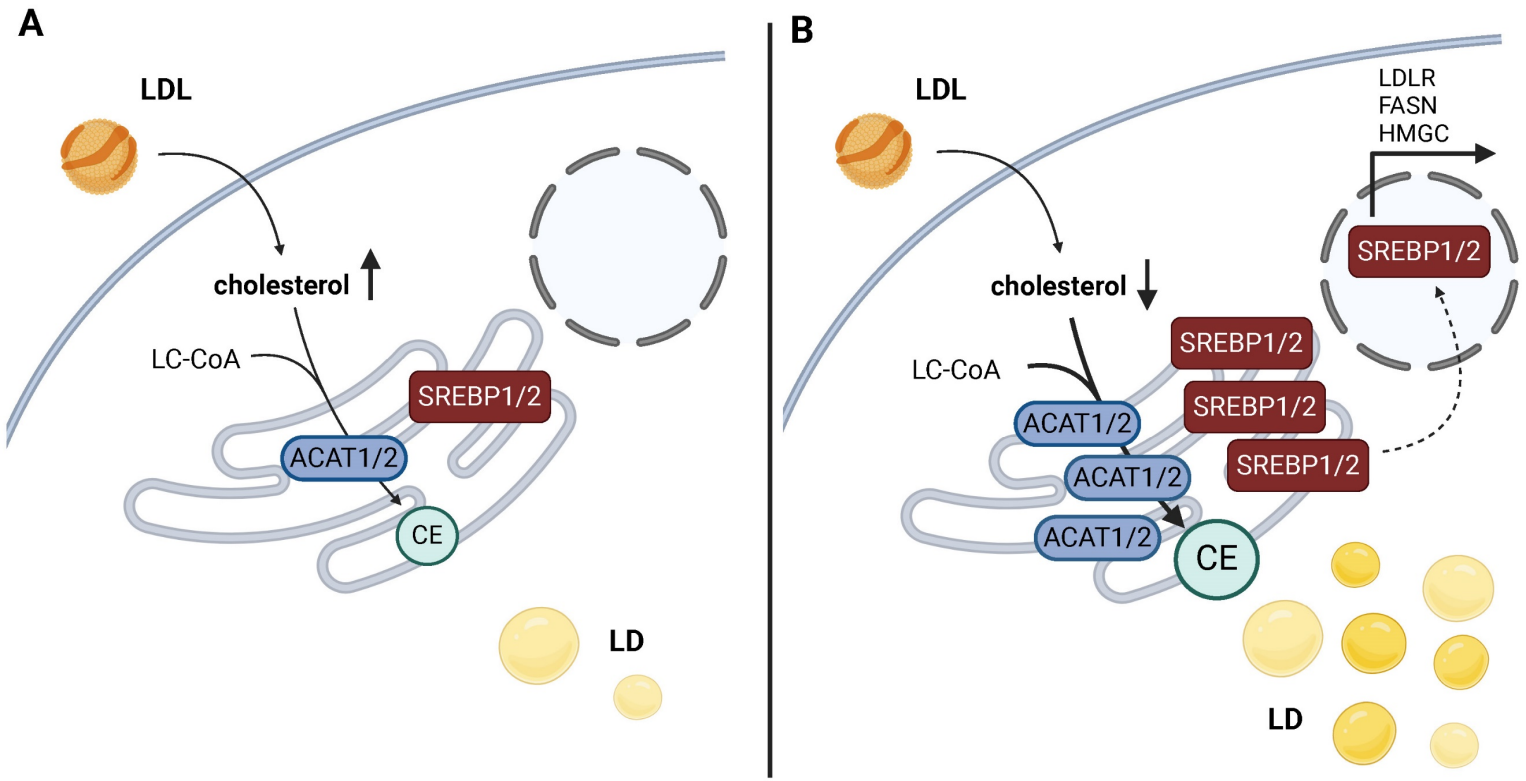
Mechanism of SREBP activation loop based on ACAT activity and cholesterol levels in cancer. **a**) High levels of cholesterol keep levels of maturated SREBP low. **b**) Low free cholesterol levels are crucial in the stabilization process of SREBP, a transcription factor targeting a gene battery related to cholesterol and FA handling. The esterification of free cholesterol into CE by ACAT1/2 keeps cholesterol levels low, which results in SREBP1/2 stabilization, its migration into the nucleus, and the transcription of SREBP-regulated genes. This leads to the further amplification of cholesterol synthesis and its import, and dependence on cholesterol metabolism for survival. Created with BioRender.com. *Abbreviations*: **ACAT:** acetyl-CoA acetyltransferase; **CE:** cholesteryl ester; **FASN:** fatty acid synthase; **HMGCS:** hydroxymethylglutaryl-CoA synthase; **LC-CoA:** long-chain CoA; **LD:** lipid droplets; **LDL:** low-density lipoprotein; **LDLR:** low-density lipoprotein receptor; **SREBP:** sterol regulatory element binding protein.

In addition to TGs, LDs are also formed in response to enhanced CE synthesis. CEs are esterified cholesterol formed in the reaction of acyl-CoA cholesterol acyltransferases 1 and 2 (ACAT1 and ACAT2), also called sterol O-acyltransferases (SOATs). Cholesterol is synthesized through the mevalonate (isoprenoid) pathway (Fig. 1b). The precursor of *de novo* cholesterol synthesis is acetyl-CoA, which undergoes a series of transformations that ultimately lead to cholesterol. Subsequently, it reacts with FAs to form CEs as a storage form of cholesterol. Both ACAT enzymes are ER-localized integral membrane proteins. The Human Protein Atlas.org (v23.proteinatlas.org) and The Adult Genotype Tissue Expression (GTEx) Project 24 report that the expression of ACAT1 is more ubiquitous than of ACAT2, but both enzymes are mainly expressed in the liver, kidney, and skeletal muscle, while ACAT2 is also significantly expressed in neural tissues. Cholesterol synthesis and uptake (in the form of LDL) is regulated by sterol regulatory element-binding protein 1 (SREBP1) or SREBP2 (encoded by SREBF1 and SREBF2, respectively, Fig. 3).

CE synthesis is vital for maintaining LD levels, but the mechanism also involves other lipogenic pathways. Although ACAT1 and ACAT2 are not expressed in white adipose tissue 25, ACAT1 was shown to be significantly increased in adipocytes of obese mice (*ob/ob*). Interestingly, the overexpression of ACAT1/2 led to a blunted differentiation of preadipocytes *in vitro*, reduced the size of LDs, their dynamics and fusion, and also downregulated DGAT1/2 expression and TG synthesis. The induction of ACAT expression was also observed in the diet-induced obesity model, as the expression of ACAT1 was augmented in white and ACAT1/2 in brown adipose tissue 26. In this case, the inhibition of ACAT1 with avasimibe suppressed *de novo* FA synthesis and TG content during adipogenesis, and impaired the adipogenic transcription program. Interestingly, this effect was more evident in the late stages of adipogenesis, which might imply the effect of an impaired SREBP1-regulated gene expression program in response to ACAT1 inhibition.

In cancer tissues, CE synthesis has been repeatedly shown to constitute a straightforward mechanism of maintaining low levels of free cholesterol in the cytosol, promoting SREBP1/2 maturation and expression of the mevalonate pathway components and the LDL receptor (Fig. 3a). In principle, the upregulation of ACAT1 leads to LD accumulation in response to CE synthesis (Fig. 3b). This results in a reduction in free cholesterol levels and subsequent activation of SREBP signaling (so-called cholesterol feedback inhibition of SREBPs), upregulation of the mevalonate pathway, and, consequently, a metabolic dependence on cholesterol synthesis 27.

The activity of ACAT1 in pancreatic carcinoma was shown to be upregulated after the inactivation of tumor protein 53 (TP53) 27 or phosphatase and tensin homolog (PTEN) 28. Both studies demonstrated that the inhibition of ACAT1 suppresses cancer progression and metastasis in xenograft models 27, 28. Furthermore, the inhibition of ACAT1 leads to an enhanced expression of glucose-regulated protein 78 (GRP78, also known as heat shock protein A5), an ER-stress marker, and cell death 28. Another report also showed an ACAT1-dependent CE-rich LD accumulation in prostate cancer, specifically in cases with deleted PTEN and an activated phosphoinositide 3-kinase / protein kinase B / mechanistic target of rapamycin (PI3K/AKT/mTOR) axis 3. Also in this case, the pharmacological inhibition of ACAT1 (but not DGAT1) alleviated tumor growth. Li 2023 29 confirmed the model of CE formation as beneficial for cell growth and invasion *in vitro*, but also emphasized the role of FA availability in the process. Vice versa, abrogating ACAT1 activity can inhibit prostate cancer growth by elevating free cholesterol levels, and downregulating expression levels of SREBP and LDL receptor 3. In accordance, a similar observation was made for liver cancer-bearing TP53 deletion; the loss of p53 signaling suppresses ATP-binding cassette transporter A1 (ABCA1, a gene encoding for the cholesterol transporter), which promoted SREBP2 maturation and the upregulation of cholesterol synthesis 30. This mechanism renders TP53-deleted tumors in the liver vulnerable to inhibition by statins, as demonstrated in xenografted mice.

Beyond handling cholesterol levels in the cell, SREBP1-dependent regulation of transforming growth factor beta (TGF-β) expression, TGF-β receptor activation, induction of a canonical mothers against decapentaplegic homologs 2/3 (SMAD2/3) effector cascade in pancreatic cancer 31, epithelial-mesenchymal transition activation, and induction of E2F transcription factor 1 (E2F1) expression 32 have been demonstrated. In summary, LD biogenesis in the context of CE metabolism is a key mechanism of maintaining SREBP1/2 activation in a similar manner to oncogenic events. Likewise with TG synthesis via DGAT1, the deposition of CEs into LDs could protect from the harmful lipotoxic effects of free cholesterol or FAs.

## 4. Formation of lipid droplets in response to limited FA import into mitochondria

In episodes of acutely elevated cytosolic FAs, such as reinforced autophagy or enhanced FA uptake from the extracellular space, LDs could sequester FAs. Although the activity of DGAT1 appears to be crucial in re-routing FAs into the LD compartment, LD biogenesis might be also a consequence of inhibited mitochondrial FA import. We have recently demonstrated that mitochondria, via the regulation of FA import, are responsible for the formation of LDs. It remains to be established which mechanisms rule the intracellular FA trafficking between organelles and how its specific requirements, metabolic or structural, are controlled. In this chapter, we discuss several aspects of LD formation in terms of lipotoxicity, mitochondrial metabolism, and inter-organelle crosstalk.

### 4.1 LDs and cytosolic lipotoxicity

To understand the role of LDs as a buffering organelle, it is essential to consider that the mechanisms of cytosolic toxicity arise from free FAs or their activated forms, particularly in the context of ferroptosis activation. Redirecting non-esterified FAs and PUFAs into LDs during the onset of autophagy provides a defense against membrane peroxidation, a recognized trigger of ferroptosis. Activated FAs can be incorporated into PLs, and PUFA-containing PLs are often subjected to peroxidation. Under normal conditions, lipid peroxides are converted into non-reactive lipid alcohols by glutathione peroxidase 4 (GPX4), a reduced glutathione-dependent lipid hydroperoxidase. GPX4 inactivation promotes lipid peroxide accumulation and subsequent ferroptosis. The formation of LDs lowers the availability of FAs for PL synthesis or mitochondrial import.

Besides GPX4, ferroptosis activation depends on the composition of the FA pool in the cell, particularly the ratio of PUFAs to monounsaturated fatty acids (MUFAs). Supplementation with MUFAs, but not PUFAs, suppresses sensitivity to erastin, a ferroptosis-inducing agent, because MUFAs repopulate PLs, as demonstrated *in vitro* 33. Interestingly, the protective effect of MUFAs is dependent on the activity of acyl-CoA synthetase long-chain family member 3 (ACSL3), the expression of which sensitizes cells to GPX4 inhibitors. These findings were confirmed in renal cell carcinoma, in which ACSL3 deletion reduced orthotopic tumor growth 34. In this model, treatment with exogenous PUFAs augmented susceptibility to erastin toxicity, particularly with linoleic and arachidonic acids (PUFAs), but not with oleic acid (OA, MUFA). Unless shuttled into LDs, non-esterified PUFAs enter the reaction of arachidonate 5-lipoxygenase (ALOX5), which further conveys the toxic effects of PUFAs and promotes cell death. The hypothesis that PUFAs are the source of cytosolic lipotoxicity was also supported by Jarc *et al.*, who showed that the inhibition of DGAT1 leads to cell death in docosahexaenoic acid-loaded cells 35, further affirming the role of LDs in mitigating PUFA-associated toxicity. Thus, current knowledge indicates that the mechanism of FA-induced cytotoxicity could be via sensitization to ferroptosis and underscores the preventive role of LDs in this process, as has also been demonstrated in β-cells 36.

### 4.2 LD biogenesis prevents mitochondrial lipotoxic dysfunction

**Figure 4:**
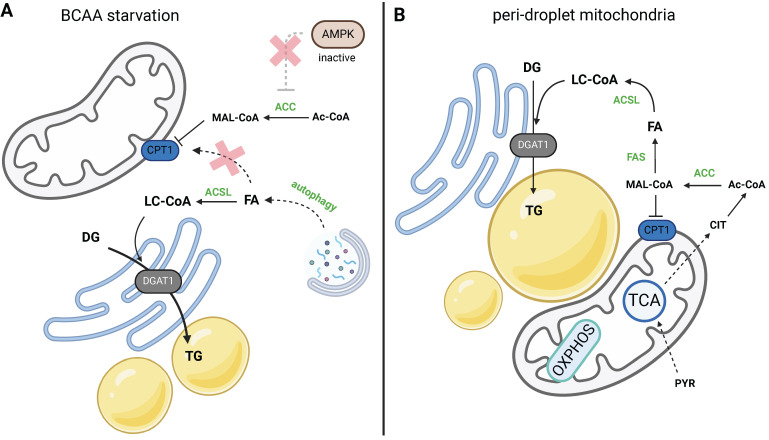
Two models of LD biogenesis due to CPT1 inhibition. **a)** During amino acid starvation inhibition, ACC is activated due to low AMPK activity, and malonyl-CoA (MAL-CoA) inhibits CPT1. FAs released by activated autophagy are not utilized in β-oxidation, but are redirected to TG synthesis. **b)** Peridroplet mitochondria contribute to *de novo* FA synthesis via citrate export and suppressed β-oxidation upon CPT1 inhibition. Created with BioRender.com. *Abbreviations*: **ACC:** acetyl-CoA carboxylase; **Ac-CoA:** acetyl-CoA; **ACSL:** long-chain-fatty-acid-CoA ligase; **AMPK:** AMP-activated protein kinase; **BCAA:** branched-chain amino acid; **CIT:** citrate;** CPT1:** carnitine palmitoyltransferase 1; **DG:** diacylglycerol; **DGAT1:** diacylglycerol O-acyltransferase; **FA:** fatty acid; **FAS:** fatty acid synthase; **MAL-CoA:** malonyl-CoA; **OXPHOS:** oxidative phosphorylation; **PYR:** pyruvate; **TCA:** citric acid cycle; **TG:** triacylglycerol.

During periods of nutrient scarcity, cells decompose their organelles to gain macromolecules and recycle them. It is well documented by now that autophagy culminates in the accumulation of LDs (Fig. 4a). In a spectrum of cultured cells of non-adipose origin, it has been shown that amino acid starvation induces autophagy and subsequent LD biogenesis 37-39. It was also conclusively demonstrated that amino acid starvation-induced autophagy could be conveyed through mammalian target of rapamycin complex 1 (MTORC1) activity 38; the constitutive activation of MTORC1 blocks amino acid starvation-induced LD biogenesis, and treatment with MTORC1 inhibitor can activate LD biogenesis in the presence of amino acids. Besides, there are other mechanisms regulating lipid accessibility in response to nutrient starvation, for instance, via promoting lipophagy and maintaining interaction between mitochondria and LDs 40. There is also an agreement that it is DGAT1, but not DGAT2, that catalyzes LD buildup in the onset of autophagy 38, 41. LD biogenesis by way of DGAT1 neutralizes the toxicity induced by the outburst of free FAs, and the complete inhibition of DGAT1/2 activity promotes toxicity in starving cells 38.

Besides starvation-induced autophagy, the presence of LDs has been identified in several models of mitochondrial dysfunction. However, owing to the spectrum of distinct models and approaches, we have no unifying mechanism explaining the phenomenon. LD accumulation has been reported with myocardial ischemia, loss of mtDNA, sepsis-induced hepatocyte, and kidney dysfunction 42. Recent work observed LD accumulation in mouse models of cytochrome c oxidase subunit 10 (COX10), succinate dehydrogenase subunit A (SDHA), and mitochondrial aspartyl-tRNA synthetase 2 (DARS2) intestinal epithelial cell-specific KO models 43. In the DARS2 KO model, enhanced TG synthesis was positively associated with activating transcription factor 4 (ATF4) activation, which is a recognized stress-induced pathway. Dysregulated TG synthesis in enterocytes results in impaired lipid handling and excretion across intestinal cells. Long *et al.* 44 reported that deferiprone treatment induced DGAT1-specific TG induction in cells derived from retinal pigment epithelia (RPE), but the mechanism of such induction, or the source of FAs (endogenous, exogenous, or rewired) was not clarified. In another RPE model, peroxisome proliferator-activated receptor gamma coactivator 1-alpha (PGC-1α) KO was associated with LD biogenesis due to enhanced cholesterol synthesis and esterification, but not TG synthesis (even though this was not assessed by pharmacological or genetic inhibition) 45. Work is still being done to determine whether there is a universal mechanism that leads to LD formation when mitochondrial function is inhibited.

Given that LDs are formed during autophagy and/or nutrient scarcity, there is, of course, the question of what the benefit is of FAs being channeled into TG synthesis instead of its direct oxidation and ATP production. Using a pulse-chase assay, Rambold 39 showed that after autophagy, FAs are deposited into LDs and mitochondrial FA oxidation follows their deposition into LDs and lipolytic release. Excessive FAs are released from cells. So, FAs liberated during autophagy are not directly imported into mitochondria, but that step is always preceded by LD formation. In accordance with this, our data showed that upon branched-chain amino acid (BCAA) starvation, the mitochondrial import of FAs is blocked, hence LDs are formed as a consequence of the blockade 41 (Fig. 4a). This implies the existence of a mechanism regulating the mitochondrial oxidation of FAs versus other FA-utilizing pathways, and suggests that LD formation could be secondary to inhibited mitochondrial import. So, during periods of FA bursts in the cytosol, mitochondria hinder FA uptake, which leads to the active redirection of FAs to TG synthesis.

The ability to limit FA import into mitochondria might be crucial for preventing mitochondrial lipotoxicity. Our data showed that DGATi is more harmful to cells that are able to import FAs into mitochondria than to cells unable to import FAs 41, which supports the idea of mitochondrial lipotoxic damage upon FA overload. However, whether mitochondrial lipotoxicity is a result of the nature of certain lipid intermediates or levels of ROS produced during β-oxidation remains to be determined. Nguyen suggested that it is the accumulation of long-chain carnitines (LC-CARs), imported into mitochondria that are the toxic agents causing mitochondria lipotoxic dysfunction, and which might be prevented by LD formation 38. This was based on the observation that carnitine palmitoyltransferase 1 (CPT1) inhibition restored the mitochondrial membrane potential in DGAT1-inhibited cells. However, this finding does not identify the specific toxicity mechanism, but highlights that the prevention of FA import and concomitant LD formation protects starving cells from an unspecified toxicity within the mitochondrial compartment. On the other hand, the study of Rambold 39 implies that the nature of FAs released by autophagy versus the nature of FAs released from LDs by lipolysis might be qualitatively different, for instance by the FA's length or its level of saturation.

In contrast to cytosolic toxicity, lipotoxic effects in mitochondria might be preferentially conveyed by saturated FAs, but it is not clear what the actual nature of the mitochondrial lipotoxic dysfunction is, i.e. a substrate overload beyond the capacity of FA oxidation, ROS burst, or the capacity of the electron transport chain. Treatment with saturated FAs, such as palmitic acid (PA), has been described to cause highly lipotoxic effects, mitochondrial DNA damage, and trigger apoptosis 46. In differentiated myotubes, the effect of various FAs on mitochondrial function 47 was clearly different; saturated FAs, i.e. PA and stearic acid decreased respiratory capacity, lowered membrane potential, and induced mitochondrial fragmentation, which was all counteracted by supplementation with OA, linoleic, or palmitoleic acid. Interestingly, PA treatment of chondrocytes was associated with increased superoxide production in the mitochondrial compartment with no observed LD biogenesis, in contrast to OA, which was more potent at inducing mitochondrial respiration as well as LD biogenesis 48. Moreover PA but not OA treatment also led to ATP depletion in chondrocytes 48. Thus, this raises the question of whether the metabolic path of an individual FA species rather than its saturation level defines its biological function. The comparison of the metabolic fate of OA and PA was also performed in myotubes 49, which usually undergo apoptosis after PA treatment *in vitro*. In myotubes, in contrast to chondrocytes, PA was more potently incorporated into TGs than OA, and it was less likely to be targeted for oxidation than OA. Using a constitutively active mutant of CPT1, it was demonstrated that mitochondrial PA import and oxidation do not negatively affect mitochondrial function and do not induce apoptosis, so there might be a different aspect of FA biology that is detrimental for cells, such as its redirection to ceramide synthesis 49, as was also suggested by 22. Contrary to these findings, we have previously demonstrated that the prevention of FA import into mitochondria and its oxidation during BCAA starvation can avert mitochondrial lipotoxic dysfunction in pancreatic cancer cells 41. However, we acknowledge that tissue specificity can be crucial and intracellular FA fate can be dictated by the expression pattern of ACSL or CPT enzyme viariants and their substrate preferences toward the respective FA species.

### 4.3 CPT1 in regulating FA oxidation and LD formation

The discussed evidence points to the existence of inter-organelle FA trafficking mechanism that ultimately regulates LD formation and FA oxidation. Molecular mechanisms favoring one or another situation might be inferred from the lipidomics landscape of activated FA intermediates. Several lines of evidence point to an inverse correlation between LC-CARs and TGs; the inhibition of DGAT1 induces an accumulation of LC-CARs 21, 22, 41, while CPT1 inhibition attenuates LC-CAR levels as opposed to a synthesis of TGs. So, there is an actual reciprocity between FA oxidation and TG synthesis, and the redistribution of FAs between storage and oxidation depends on the levels of LC-CARs, which point to the crucial involvement of CPT1 in the regulation of FA trafficking.

The activity of CPT1 is arranged by AMP-activated protein kinase (AMPK) signaling, which could potentially dictate FA oxidation based on the actual energy requirements. The regulation of CPT1 by malonyl-CoA is an elegant way to ensure the reciprocity of FA oxidation and FA synthesis (Fig. 4). AMPK signaling toward CPT1 activation is enabled by acetyl-CoA carboxylase (ACC); malonyl-CoA is a product of ACC, and ACC itself is a target of AMPK. As an illustrative example, upon glucose starvation, ACC is deactivated by phosphorylation, which results in decreased levels of malonyl-CoA, CPT1 derepression, and subsequent activation of mitochondrial FA oxidation 50. KO of ACC2 in mice reportedly enhanced FA oxidation and reduced TG content in the liver 51. Furthermore, this paradigm was confirmed for the LD-associated mitochondria (LDM) in the liver 52. LDM is the population of mitochondria that cluster in the proximity of LDs, physically interact with LDs, hence the isolation protocol of LDM requires specific approach. It was demonstrated, that in LDM, CPT1 activity depends on the positive phosphorylation status of ACC2, which in turn maintains higher β-oxidation in LDM than “cytosolic” mitochondria. On the other hand, this mechanism also enables LD expansion during the switch to diet-induced non-alcoholic fatty liver disease, which promotes a decrease in ACC2 phosphorylation and suppression of CPT1 activity 52.

Another level of AMPK signaling interfering with FA trafficking between LDs and mitochondria was discovered by Ouyang 2023 53, demonstrating that AMPK promotes the mobilization of FAs from LD in favor of β-oxidation. Using a model of serum starvation in mouse myotubes, it was shown that the knockdown of AMPK suppressed FA trafficking into mitochondria, which is in line with 41. That work also demonstrated a reciprocity between FA trafficking between mitochondria and LDs, given that serum-starved cells imported FAs into mitochondria while serum-fed myoblasts imported FAs into LDs. The mechanism encompasses the recruitment of GPTase Rab8A into the mitochondrial membrane upon phosphorylation by AMPK, causing tethering of the mitochondrial outer membrane with LDs due to the interaction of perilipin 5 with Rab8A. The complex also includes adipose triglyceride lipase (ATGL) recruitment and promotes TG lipolysis, FA mobilization, and the transfer of released FAs from LDs to mitochondria for oxidation.

Besides CPT1, AMPK also regulates the motility of LDs itself. In the absence of glucose, LDs are decomposed in favor of β-oxidation; LD clusters are dispersed and individual LDs are more mobile due to detyrosinated tubulin and microtubule distribution 54. Therefore, it is plausible that AMPK supports FA oxidation not only via ACC inhibition by phosphorylation, but also by increasing LD mobility and their interactions with mitochondria, which collectively enable robust FA trafficking into the mitochondria during glucose or serum starvation. In contrast, starvation of certain amino acids, such as leucine, valine, isoleucine, and glutamine (and possibly not limited to those) impairs FA import into mitochondria, facilitated by blunted AMPK signaling 41.

Current knowledge regarding CPT1's sensitivity to malonyl-CoA and mitochondrial network architecture was recently extended beyond AMPK signaling; curved or fragmented mitochondria are insensitive to malonyl-CoA and exhibit enhanced β-oxidation, as demonstrated using several genetic models 55. This is an important finding, showing that the level of mitochondrial fragmentation truly defines functional mitochondrial subpopulations in terms of substrate utilization. Nevertheless, CPT1's sensitivity to malonyl-CoA based on the shape of the mitochondrial membrane has been already shown by Frigini 56, who compared planar and curved membranes to study the molecular dynamics of CPT1. So, the level of elongation or fragmentation of the mitochondrial network mechanistically dictates the utilization of FAs and delineates functional subpopulations of mitochondria. On the other hand, another line of evidence points to the relevance of CPT1 activity in the build-up of LDs 57 analogous to 52; the study demonstrated that a certain subpopulation of mitochondria, termed peridroplet mitochondria (Fig. 4b), exhibit higher substrate oxidation, but not PA oxidation. In this case, mitochondria via citrate export and malonyl-CoA synthesis regulated CPT1 activation status and hence FA import into mitochondria, or their deposition into LDs.

## 5. Conclusions

Recent advancements in liquid chromatography-mass spectrometry-based high-throughput profiling techniques and transcriptome profiling platforms offer unprecedented opportunities to explore known biological processes and interactions in greater depth. There has been a recent trend of biochemical work that have identified biological situations under which TGs accumulate in response to mitochondrial manipulations or defects. This suggests that LD formation may occur in unexplored *in vitro* and *in vivo* contexts, regulated by both genetic and metabolic factors. Beyond the established function of LDs in storing and releasing FAs, a role for them has emerged in buffering cytosolic FAs, thereby mitigating their potential toxic effects. The complex interplay between LDs and cellular toxicity, particularly within the domains of mitochondrial and cytosolic compartments, represents a poorly explored avenue in contemporary cell biology.

Based on the provided information, we can summarize that both TG and CE metabolism contribute to LD biogenesis, and importantly, the factor of inter-organelle crosstalk regulating β-oxidation and LD formation has provided a new perspective on LDs and mitochondria function. In light of our discussion, we propose several questions to be addressed at the biochemical level, such as i) which FA species are deposited into LDs versus those released from LDs, and which substrates are preferred by the enzymatic machinery involved in LD formation or decomposition; ii) which factors and biochemical contexts contribute to toxicity in mitochondria and cytosol; iii) is LD accumulation a compensatory mechanism for chronic autophagy in certain cancer tissues? Unraveling the specific biochemical mechanisms that guide the deposition and lipolytic release of FAs, and deciphering the factors contributing to toxicity in both mitochondria and the cytosol should advance our fundamental understanding of cellular processes.

## Abbreviations

ABCA1: ATP-binding cassette transporter A1
ACAT: acetyl-CoA acetyltransferase
ACC: acetyl-CoA carboxylase
Ac-CoA: acetyl-coenzyme A
ACSL3: long-chain acyl-CoA synthase 3
AMPK: AMP-activated protein kinase
p-AMPK: phosphorylated AMPK
ATF4: activating transcription factor 4
ATGL: adipose triglyceride lipase
BCAA: branched-chain amino acids
CACT: carnitine-acylcarnitine translocase
CE: cholesteryl esters
COX10: cytochrome c oxidase subunit 10
CPT: carnitine palmitoyltransferase
DG: diacylglycerol
DGAT: acyl-CoA:diacylglycerol acyltransferase
DGATi: DGAT inhibition
E2F1: E2F transcription factor 1
ER: endoplasmic reticulum
FA: fatty acid
G3P: glycerol-3-phosphate
GBM: glioblastoma multiform
GRP78: glucose-regulated protein 78
GPX4: glutathione peroxidase 4
HDL: high-density lipoprotein
HFD: high-fat diet
KO: knockout
LC-CAR: long-chain carnitines
LC-CoA: long-chain coenzyme A
LD: lipid droplets
LDL: low density lipoprotein
LDM: LD-associated mitochondria
MEF: mouse embryonal fibroblast
MTORC1: mammalian target of rapamycin complex 1
MUFA: monounsaturated fatty acid
OA: oleic acid
PA: palmitic acid
PC: phosphatidylcholine
PE: phosphatidyletanolamin
PI3K/AKT/mTOR: phosphoinositide 3-kinase / protein kinase B / mechanistic target of rapamycin
PGC1-α: peroxisome proliferator-activated receptor gamma coactivator 1-alpha
PL: phospholipid
PS: phosphatidylserine
PTEN: phosphatase and tensin homolog
PUFA: polyunsaturated fatty acids
ROS: reactive oxygen species
RPE: retinal pigment epithelia
SC-CAR: short-chain carnitines
SC-CoA: short-chain coenzyme A
SDHA: succinate dehydrogenase subunit A
SMAD2: mothers against decapentaplegic homolog 2
SREBP: sterol regulatory element binding protein
TG: triacylglycerol
TGF-β: transforming growth factor beta
TP53: tumor protein 53

## References

[1] Accioly MT, Pacheco P, Maya-Monteiro CM, Carrossini N, Robbs BK, Oliveira SS (2008). Lipid bodies are reservoirs of cyclooxygenase-2 and sites of prostaglandin-E2 synthesis in colon cancer cells. Cancer Res.

[2] Qiu B, Ackerman D, Sanchez DJ, Li B, Ochocki JD, Grazioli A (2015). HIF2α-Dependent Lipid Storage Promotes Endoplasmic Reticulum Homeostasis in Clear-Cell Renal Cell Carcinoma. Cancer Discov.

[3] Yue S, Li J, Lee SY, Lee HJ, Shao T, Song B (2014). Cholesteryl ester accumulation induced by PTEN loss and PI3K/AKT activation underlies human prostate cancer aggressiveness. Cell Metab.

[4] Hultsch S, Kankainen M, Paavolainen L, Kovanen R-M, Ikonen E, Kangaspeska S (2018). Association of tamoxifen resistance and lipid reprogramming in breast cancer. BMC Cancer.

[5] Rossmeislová L, Gojda J, Smolková K (2021). Pancreatic cancer: branched-chain amino acids as putative key metabolic regulators?. Cancer and Metastasis Reviews.

[6] Choudhary V, El Atab O, Mizzon G, Prinz WA, Schneiter R (2020). Seipin and Nem1 establish discrete ER subdomains to initiate yeast lipid droplet biogenesis. The Journal of cell biology.

[7] Wang L, Qian H, Nian Y, Han Y, Ren Z, Zhang H (2020). Structure and mechanism of human diacylglycerol O-acyltransferase 1. Nature.

[8] Kuerschner L, Moessinger C, Thiele C (2008). Imaging of Lipid Biosynthesis: How a Neutral Lipid Enters Lipid Droplets. Traffic.

[9] Wilfling F, Wang H, Haas JT, Krahmer N, Gould TJ, Uchida A (2013). Triacylglycerol synthesis enzymes mediate lipid droplet growth by relocalizing from the ER to lipid droplets. Dev Cell.

[10] Lee J, Ridgway ND (2020). Substrate channeling in the glycerol-3-phosphate pathway regulates the synthesis, storage and secretion of glycerolipids. Biochimica et Biophysica Acta (BBA) - Molecular and Cell Biology of Lipids.

[11] Harris CA, Haas JT, Streeper RS, Stone SJ, Kumari M, Yang K (2011). DGAT enzymes are required for triacylglycerol synthesis and lipid droplets in adipocytes. J Lipid Res.

[12] Yen CL, Stone SJ, Koliwad S, Harris C, Farese RV Jr (2008). Thematic review series: glycerolipids. DGAT enzymes and triacylglycerol biosynthesis. J Lipid Res.

[13] Chitraju C, Fischer AW, Ambaw YA, Wang K, Yuan B, Hui S (2023). Mice lacking triglyceride synthesis enzymes in adipose tissue are resistant to diet-induced obesity. Elife.

[14] McLelland GL, Lopez-Osias M, Verzijl CRC, Ellenbroek BD, Oliveira RA, Boon NJ (2023). Identification of an alternative triglyceride biosynthesis pathway. Nature.

[15] Smith SJ, Cases S, Jensen DR, Chen HC, Sande E, Tow B (2000). Obesity resistance and multiple mechanisms of triglyceride synthesis in mice lacking Dgat. Nat Genet.

[16] Stone SJ, Myers HM, Watkins SM, Brown BE, Feingold KR, Elias PM (2004). Lipopenia and skin barrier abnormalities in DGAT2-deficient mice. J Biol Chem.

[17] Chitraju C, Walther TC, Farese RV Jr (2019). The triglyceride synthesis enzymes DGAT1 and DGAT2 have distinct and overlapping functions in adipocytes. J Lipid Res.

[18] Villanueva CJ, Monetti M, Shih M, Zhou P, Watkins SM, Bhanot S (2009). Specific role for acyl CoA:Diacylglycerol acyltransferase 1 (Dgat1) in hepatic steatosis due to exogenous fatty acids. Hepatology (Baltimore, Md).

[19] Gluchowski NL, Gabriel KR, Chitraju C, Bronson RT, Mejhert N, Boland S (2019). Hepatocyte Deletion of Triglyceride-Synthesis Enzyme Acyl CoA: Diacylglycerol Acyltransferase 2 Reduces Steatosis Without Increasing Inflammation or Fibrosis in Mice. Hepatology (Baltimore, Md).

[20] Hung YH, Carreiro AL, Buhman KK (2017). Dgat1 and Dgat2 regulate enterocyte triacylglycerol distribution and alter proteins associated with cytoplasmic lipid droplets in response to dietary fat. Biochimica et biophysica acta Molecular and cell biology of lipids.

[21] Wilcock DJ, Badrock AP, Wong CW, Owen R, Guerin M, Southam AD (2022). Oxidative stress from DGAT1 oncoprotein inhibition in melanoma suppresses tumor growth when ROS defenses are also breached. Cell Rep.

[22] Cheng X, Geng F, Pan M, Wu X, Zhong Y, Wang C (2020). Targeting DGAT1 Ameliorates Glioblastoma by Increasing Fat Catabolism and Oxidative Stress. Cell Metab.

[23] Mitra R, Le TT, Gorjala P, Goodman OB Jr (2017). Positive regulation of prostate cancer cell growth by lipid droplet forming and processing enzymes DGAT1 and ABHD5. BMC Cancer.

[24] Lonsdale J, Thomas J, Salvatore M, Phillips R, Lo E, Shad S (2013). The Genotype-Tissue Expression (GTEx) project. Nature Genetics.

[25] Xu Y, Du X, Turner N, Brown AJ, Yang H (2019). Enhanced acyl-CoA:cholesterol acyltransferase activity increases cholesterol levels on the lipid droplet surface and impairs adipocyte function. J Biol Chem.

[26] Zhu Y, Chen C-Y, Li J, Cheng J-X, Jang M, Kim K-H (2018). In vitro exploration of ACAT contributions to lipid droplet formation during adipogenesis. Journal of Lipid Research.

[27] Oni TE, Biffi G, Baker LA, Hao Y, Tonelli C, Somerville TDD (2020). SOAT1 promotes mevalonate pathway dependency in pancreatic cancer. The Journal of experimental medicine.

[28] Li J, Gu D, Lee SSY, Song B, Bandyopadhyay S, Chen S (2016). Abrogating cholesterol esterification suppresses growth and metastasis of pancreatic cancer. Oncogene.

[29] Li Y, Amrutkar M, Finstadsveen AV, Dalen KT, Verbeke CS, Gladhaug IP (2023). Fatty acids abrogate the growth-suppressive effects induced by inhibition of cholesterol flux in pancreatic cancer cells. Cancer Cell Int.

[30] Moon SH, Huang CH, Houlihan SL, Regunath K, Freed-Pastor WA, Morris JPt (2019). p53 Represses the Mevalonate Pathway to Mediate Tumor Suppression. Cell.

[31] Gabitova-Cornell L, Surumbayeva A, Peri S, Franco-Barraza J, Restifo D, Weitz N (2020). Cholesterol Pathway Inhibition Induces TGF-β Signaling to Promote Basal Differentiation in Pancreatic Cancer. Cancer Cell.

[32] Xiong K, Wang G, Peng T, Zhou F, Chen S, Liu W (2021). The cholesterol esterification inhibitor avasimibe suppresses tumour proliferation and metastasis via the E2F-1 signalling pathway in prostate cancer. Cancer Cell International.

[33] Magtanong L, Ko PJ, To M, Cao JY, Forcina GC, Tarangelo A (2019). Exogenous Monounsaturated Fatty Acids Promote a Ferroptosis-Resistant Cell State. Cell Chem Biol.

[34] Klasson TD, LaGory EL, Zhao H, Huynh SK, Papandreou I, Moon EJ (2022). ACSL3 regulates lipid droplet biogenesis and ferroptosis sensitivity in clear cell renal cell carcinoma. Cancer Metab.

[35] Jarc E, Kump A, Malavašič P, Eichmann TO, Zimmermann R, Petan T (2018). Lipid droplets induced by secreted phospholipase A2 and unsaturated fatty acids protect breast cancer cells from nutrient and lipotoxic stress. Biochimica et Biophysica Acta (BBA) - Molecular and Cell Biology of Lipids.

[36] Thomas P, Arden C, Corcoran J, Hacker C, Welters HJ, Morgan NG (2022). Differential routing and disposition of the long-chain saturated fatty acid palmitate in rodent vs human beta-cells. Nutrition & Diabetes.

[37] Jusović M, Starič P, Jarc Jovičić E, Petan T (2023). The Combined Inhibition of Autophagy and Diacylglycerol Acyltransferase-Mediated Lipid Droplet Biogenesis Induces Cancer Cell Death during Acute Amino Acid Starvation. Cancers.

[38] Nguyen TB, Louie SM, Daniele JR, Tran Q, Dillin A, Zoncu R (2017). DGAT1-Dependent Lipid Droplet Biogenesis Protects Mitochondrial Function during Starvation-Induced Autophagy. Developmental Cell.

[39] Rambold AS, Cohen S, Lippincott-Schwartz J (2015). Fatty acid trafficking in starved cells: regulation by lipid droplet lipolysis, autophagy, and mitochondrial fusion dynamics. Dev Cell.

[40] Cusenza VY, Bonora E, Amodio N, Frazzi R (2022). Spartin: At the crossroad between ubiquitination and metabolism in cancer. Biochim Biophys Acta Rev Cancer.

[41] Gotvaldová K, Špačková J, Novotný J, Baslarová K, Ježek P, Rossmeislová L (2024). BCAA metabolism in pancreatic cancer affects lipid balance by regulating fatty acid import into mitochondria. Cancer & Metabolism.

[42] Lee SJ, Zhang J, Choi AM, Kim HP (2013). Mitochondrial dysfunction induces formation of lipid droplets as a generalized response to stress. Oxidative medicine and cellular longevity.

[43] Moschandrea C, Kondylis V, Evangelakos I, Herholz M, Schneider F, Schmidt C (2024). Mitochondrial dysfunction abrogates dietary lipid processing in enterocytes. Nature.

[44] Long M, Sanchez-Martinez A, Longo M, Suomi F, Stenlund H, Johansson AI (2022). DGAT1 activity synchronises with mitophagy to protect cells from metabolic rewiring by iron depletion. The EMBO Journal.

[45] Zhou S, Taskintuna K, Hum J, Gulati J, Olaya S, Steinman J (2024). PGC-1α repression dysregulates lipid metabolism and induces lipid droplet accumulation in retinal pigment epithelium. Cell Death Dis.

[46] Yuzefovych L, Wilson G, Rachek L (2010). Different effects of oleate vs. palmitate on mitochondrial function, apoptosis, and insulin signaling in L6 skeletal muscle cells: role of oxidative stress. Am J Physiol Endocrinol Metab.

[47] Nisr RB, Shah DS, Hundal HS (2020). Mono- and Polyunsaturated Fatty Acids Counter Palmitate-Induced Mitochondrial Dysfunction in Rat Skeletal Muscle Cells. Cellular physiology and biochemistry: international journal of experimental cellular physiology, biochemistry, and pharmacology.

[48] Vázquez-Mosquera ME, Fernández-Moreno M, Cortés-Pereira E, Relaño S, Dalmao-Fernández A, Ramos-Louro P (2021). Oleate Prevents Palmitate-Induced Mitochondrial Dysfunction in Chondrocytes. Front Physiol.

[49] Henique C, Mansouri A, Fumey G, Lenoir V, Girard J, Bouillaud F (2010). Increased Mitochondrial Fatty Acid Oxidation Is Sufficient to Protect Skeletal Muscle Cells from Palmitate-induced Apoptosis*. Journal of Biological Chemistry.

[50] Srivastava RA, Pinkosky SL, Filippov S, Hanselman JC, Cramer CT, Newton RS (2012). AMP-activated protein kinase: an emerging drug target to regulate imbalances in lipid and carbohydrate metabolism to treat cardio-metabolic diseases. J Lipid Res.

[51] Abu-Elheiga L, Matzuk MM, Abo-Hashema KAH, Wakil SJ (2001). Continuous Fatty Acid Oxidation and Reduced Fat Storage in Mice Lacking Acetyl-CoA Carboxylase 2. Science.

[52] Talari NK, Mattam U, Meher NK, Paripati AK, Mahadev K, Krishnamoorthy T (2023). Lipid-droplet associated mitochondria promote fatty-acid oxidation through a distinct bioenergetic pattern in male Wistar rats. Nature Communications.

[53] Ouyang Q, Chen Q, Ke S, Ding L, Yang X, Rong P (2023). Rab8a as a mitochondrial receptor for lipid droplets in skeletal muscle. Developmental Cell.

[54] Herms A, Bosch M, Reddy BJN, Schieber NL, Fajardo A, Rupérez C (2015). AMPK activation promotes lipid droplet dispersion on detyrosinated microtubules to increase mitochondrial fatty acid oxidation. Nature Communications.

[55] Ngo J, Choi DW, Stanley IA, Stiles L, Molina AJA, Chen PH (2023). Mitochondrial morphology controls fatty acid utilization by changing CPT1 sensitivity to malonyl-CoA. Embo j.

[56] Frigini EN, Barrera EE, Pantano S, Porasso RD (2020). Role of membrane curvature on the activation/deactivation of Carnitine Palmitoyltransferase 1A: A coarse grain molecular dynamic study. Biochimica et Biophysica Acta (BBA) - Biomembranes.

[57] Benador IY, Veliova M, Mahdaviani K, Petcherski A, Wikstrom JD, Assali EA (2018). Mitochondria Bound to Lipid Droplets Have Unique Bioenergetics, Composition, and Dynamics that Support Lipid Droplet Expansion. Cell Metabolism.

